# COVID-19, Necrotizing Pancreatitis, and Abdominal Compartment Syndrome: A Perfect Cytokine Storm?

**DOI:** 10.7759/cureus.17230

**Published:** 2021-08-16

**Authors:** Abdul Rahman Al Armashi, Francisco J Somoza-Cano, Kanchi Patell, Anas Al Zubaidi, Keyvan Ravakhah

**Affiliations:** 1 Internal Medicine, St. Vincent Charity Medical Center, Cleveland, USA; 2 Internal Medicine, Baptist Health North Little Rock, North Little Rock, USA

**Keywords:** covid 19, corona virus disease 2019, pancreatitis, acute necrotizing pancreatitis, adbominal compartment syndrome, cardiac tamponade, acute kidney injury, compartment syndrome

## Abstract

Coronavirus disease 2019 (COVID-19) induces a dysregulated immune response, leading to a drastic elevation of proinflammatory cytokines. This cytokine storm has the potential to aggravate any prior ongoing inflammation. Moreover, acute pancreatitis can cause local necrosis, thereby causing extensive abdominal inflammation. This condition increases the risk of abdominal compartment syndrome (ACS) and its deleterious consequences. We report the case of a 37-year-old male with a past medical history of chronic pancreatitis and alcohol use disorder who presented to the emergency department complaining of abdominal pain. Physical examination revealed a tender abdomen. Initial workup showed elevated amylase and lipase, a positive COVID-19 polymerase chain reaction (PCR) test, and elevated inflammatory markers. The patient denied any respiratory symptoms. Initial abdominal CT scan revealed mild pancreatic inflammation. The patient was admitted to the respiratory ICU and managed with fluid resuscitation and pain control. However, the patient had increasing oxygen requirements, leukocytosis, and worsening kidney function. A trans-bladder measurement of intra-abdominal pressure revealed severe ACS. Broad-spectrum antibiotics were started; however, after 72 hours of treatment, the patient had a cardiopulmonary arrest. He returned to spontaneous circulation after advanced cardiovascular life support (ACLS) protocol and intubation. A repeat CT scan of the abdomen showed necrotizing pancreatitis with a large-volume hemoperitoneum. Urgent pancreatic necrosectomy was performed with drainage of the hemoperitoneum. The patient was transferred to a long-term acute care facility for extended antibiotic therapy where he eventually recovered. This case illustrates the catastrophic consequences of necrotizing pancreatitis complicated by sepsis and ACS in a COVID-19-positive patient.

## Introduction

Coronavirus disease 2019 (COVID-19) induces a dysregulated immune response, which results in a drastic elevation of proinflammatory cytokines. This cytokine storm can exacerbate any prior ongoing inflammation in patients. Moreover, acute pancreatitis can cause local necrosis, causing extensive abdominal inflammation [1]. This condition increases the risk of abdominal compartment syndrome (ACS) and its detrimental consequences. We discuss the case of a 37-year-old male who presented with abdominal pain and was found to have COVID-19 and acute pancreatitis, which were complicated by ACS.

## Case presentation

A 37-year-old male with a past medical history of chronic pancreatitis and alcohol use disorder presented to the emergency department complaining of epigastric abdominal pain with radiation to the back associated with vomiting. The patient's vitals were stable. Physical examination revealed abdominal tenderness in the epigastric area, normal bowel sounds, and negative peritoneal signs. The initial workup showed white blood cells of 14 K/ul (reference range: 3.9-11), amylase of 264 U/L (reference range: 25-115), lipase of 5418 U/L (reference range: 73-393), creatinine level of 1.2 mg/dL at baseline, C-reactive protein (CRP) of 81 mg/L (reference range: 0-3), and lactate dehydrogenase of 378 U/L (reference range: 84-246). Additionally, it showed a positive COVID-19 polymerase chain reaction (PCR) test (Table 1).

**Table 1:** Preliminary laboratory workup WBC: white blood cells; Hgb: hemoglobin; BUN: blood urea nitrogen; GFR: glomerular filtration rate; CRP: C-reactive protein

Variables	Value	Reference range
WBC	14 K/ul	3.9-11
Hgb	15.6 g/dL	14-16.5
Platelets count	371 K/uL	140-440
Sodium	142 mmol/L	136-145
Potassium	3.6 mmol/L	3.5-5.1
Chloride	106 mmol/L	98-107
Creatinine	1.2 mg/dL	0.7-1.3
BUN	10 mg/dL	7-18
GFR	>60	>60
Amylase	264 U/L	25-115
Lipase	5418 U/L	73-393
CRP	81 mg/L	0-3
Lactate dehydrogenase	378 U/L	84-246

**Figure 1:**
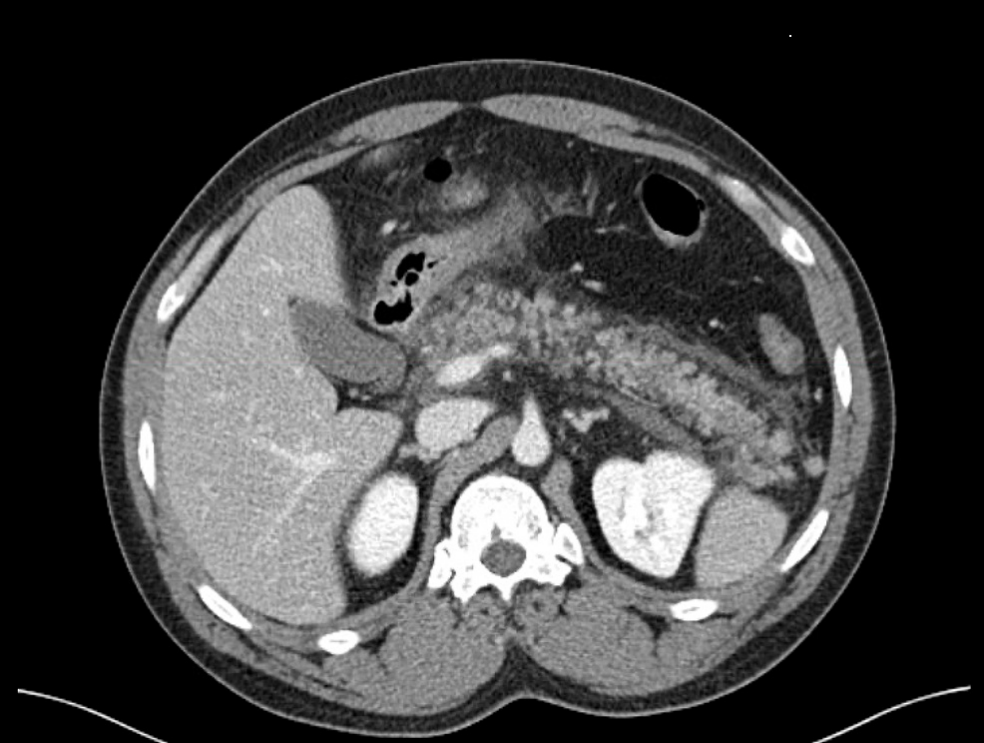
Initial CT scan of the abdomen CT scan of the abdomen with IV contrast demonstrated acute interstitial edema of the pancreas CT: computed tomography

**Figure 2:**
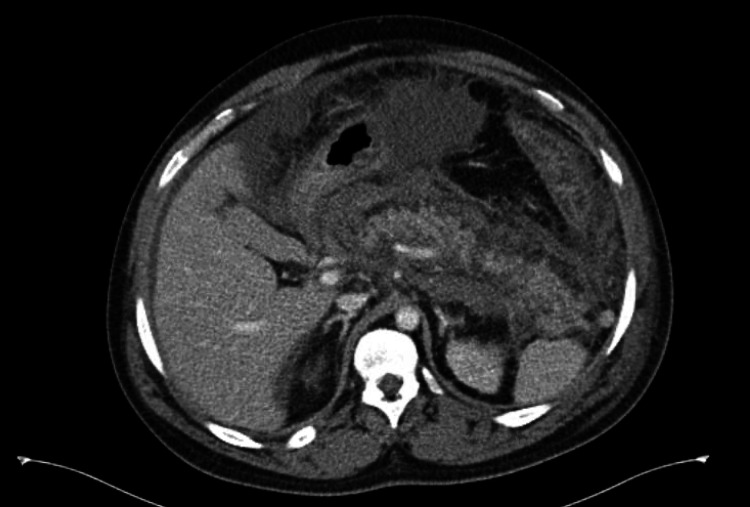
Repeated CT scan of the abdomen CT scan of the abdomen with IV contrast demonstrated necrotizing hemorrhagic pancreatitis, progression of intraabdominal edema, and hemoperitoneum CT: computed tomography

Initial abdominal CT scan revealed mild pancreatic inflammation with interstitial edema (Figure 1). The patient was admitted to the respiratory ICU and managed with fluid resuscitation and pain control. However, the patient had increasing oxygen requirements, leukocytosis, and worsening kidney function with creatinine elevation up to 6 mg/dL, and a decrease in urine output was noticed. The worsening renal function prompted suspicion for an additional pathological component; trans-bladder measurement of intra-abdominal pressure revealed severe ACS. Broad-spectrum antibiotics were started, but after 72 hours of treatment, the patient had a cardiopulmonary arrest. He returned to spontaneous circulation after advanced cardiovascular life support protocol (ACLS) and intubation. A repeat CT scan of the abdomen showed necrotizing pancreatitis with a large-volume hemoperitoneum (Figure 2). Urgent pancreatic necrosectomy was successfully performed with drainage of the hemoperitoneum. The patient was transferred to a long-term acute care facility for extended antibiotic therapy where he eventually recovered. 

## Discussion

COVID-19 affects various systems in the human body. Remarkably, the gastrointestinal system is frequently involved, including the pancreas, liver, and intestines [2,3]. COVID-19 enters the cells through the angiotensin-converting enzyme 2 (ACE2) receptor complex. Moreover, the pancreas has a high expression of ACE2, making it a target for dysregulated inflammation [1].

ACS develops as a result of recurrent elevations of pressure exceeding 20 mmHg within the abdominal cavity with evidence of organ failure [4]. It has an incidence of 15% in patients with necrotizing pancreatitis and a mortality rate of 49% [5]. Acute pancreatitis is a significant contributor to the development of intra-abdominal hypertension (IAH). Visceral edema coupled with extensive fluid resuscitation and intraabdominal inflammation-induced capillary leakage can lead to the development of ACS [6]. Additionally, the cytokine storm associated with COVID-19 can hasten the progression of acute pancreatitis course and lead to the early development of the ACS and an increase in mortality [1,7].

Furthermore, when severe acute necrotizing pancreatitis occurs, multisystem organ failure usually follows [1], including the kidneys due to the impairment of renal blood flow and glomerular filtration [8]. Additionally, in patients with ACS, the lungs are impacted due to diminished chest wall compliance caused by the diaphragm being pushed up, as seen in our patient, leading to the progressive increase in oxygen requirements [9].

According to The World Society of the Abdominal Compartment Syndrome (WSACS) guidelines, initial management includes conservative measures of frequent monitoring of bladder pressure, pain control, and a goal of restoring and assisting organ failure through hemodialysis and the use of assisted breathing. If conservative therapy fails, interventional techniques such as percutaneous catheter drainage under radiological guidance may be utilized. Decompressive laparotomy with temporary abdominal closure is advised in individuals with persistent ACS despite percutaneous treatment [10]. A retrospective study conducted in 2010 by Mentula et al. has revealed that early abdominal decompression is linked to an enhanced renal and respiratory outcome and a lower fatality rate [11].

Surgical intervention with necrosectomy is indicated in necrotizing pancreatitis patients when infected pancreatic necrosis is suspected with worsening sepsis signs and symptoms as seen in our patient [12].

## Conclusions

This case highlights the drastic consequences of necrotizing pancreatitis complicated by sepsis and ACS in a COVID-19-positive patient. ACS should be included as a differential diagnosis in the clinical course of COVID-19 and pancreatitis to prevent life-threatening complications.
